# A formula for instability-related bone loss: estimating glenoid width and redefining bare spot

**DOI:** 10.1007/s00264-024-06095-7

**Published:** 2024-02-02

**Authors:** Zhongkai Ren, Fengkun Wang, Xiaohong Huang, Jian Wang, Yingze Zhang, Tengbo Yu

**Affiliations:** 1 Department of Sports Medicine, Affiliated Hospital of Qingdao University, Qingdao, Shandong China; 2 School of Medicine, Nankai University, Tianjing, China; 3 Medical Research Center, Shandong Institute of Traumatic Orthopedics, The Affiliated Hospital of Qingdao University, Qingdao, China; 4 Department of Hand and Foot Surgery, The Affiliated Hospital of Qingdao University, Qingdao, China; 5 Department of Orthopedics, The Third Hospital of Hebei Medical University, Shijiazhuang, China; 6 Department of Orthopedics, The Affiliated Hospital of Qingdao University, Qingdao, China; 7 Department of Orthopedic Surgery, University of Health and Rehabilitation Sciences (Qingdao Municipal Hospital), Qingdao HospitalQingdao, China; 8 Institute of Sports Medicine and Health, Qingdao University, Qingdao, China

**Keywords:** Glenoid bone loss, Anterior glenohumeral instability, Cadaver-based formula, 3D-CT, Bare spot, Best-fit circle

## Abstract

**Purpose:**

The aim of the study reveals a new intuitive method for preoperatively assessing defect ratio in glenoid deficiency based on the native glenoid width and the bare spot.

**Methods:**

A linear relationship, i.e. the rh formula, between the native glenoid width (2*r*) and height (*h*) was revealed by a cadaver cohort (*n* = 204). To validate the reliability of the rh formula, 280 3D-CT images of intact glenoids were recruited. To evaluate the accuracy of rh formula in estimating glenoid defect, the 65 anterior–inferior defect models were artificially established based on the 3D-CT images of intact glenoids. Moreover, a clinically common anterior–posterior (AP) method was compared with the rh formula, to verify the technical superiority of rh formula.

**Results:**

The regression analysis indicated a linear relationship between the width and height of intact glenoid: 2*r* = 0.768 × *h* − 1.222 mm (*R*^2^ = 0.820, *p* < 0.001). An excellent reliability was found between the formula prediction and model width (*ICC* = 0.911, *p* = 0.266). An excellent agreement was found between the predicted values and model parameters (glenoid width, *ICC*_rh_ = 0.967, *p*_rh_ = 0.778; defect ratio, *p*_rh_ = 0.572, *ICC*_rh_ = 0.997). And, it is of higher accuracy compared to the AP method (glenoid width, *ICC*_AP_ = 0.933, *p*_AP_ = 0.001; defect ratio, *ICC*_AP_ = 0.911, *p*_AP_ = 0.033).

**Conclusion:**

Applying the cadaver-based formula on 3D-CT scans accurately predicts native glenoid width and redefines bare spot for preoperatively determining glenoid bone loss.

**Supplementary Information:**

The online version contains supplementary material available at 10.1007/s00264-024-06095-7.

## Introduction

The glenoid bone loss, i.e. bony Bankart lesion, has been seen in 20% of the first dislocation and 90% of the recurrent dislocation [1]. Hence, an accurate estimation of glenoid defect helps surgeons determine whether patients should undergo arthroscopic Bankart repair or bone block procedures [2]. A high risk of shoulder dislocation during 30 activities of daily living has been associated with a Bankart repair when the anterior–inferior defect corresponds to 16% of the glenoid width [2]; on this particular occasion, a bone graft operation is recommended. Glenoid bone loss is measured preoperatively using the best-fit circle which is based on the intact glenoid with the native glenoid width as the diameter and the bare spot at the centre of the intact glenoid fossa [3]. Though many methods have been developed to estimate the defect ratio in glenoid deficiency [4], including the anterior–posterior (AP) method, bias exists in locating the approximate best-fit circle in the defect glenoid [5], and no consensus has been reached [6].

**Fig. 1:**
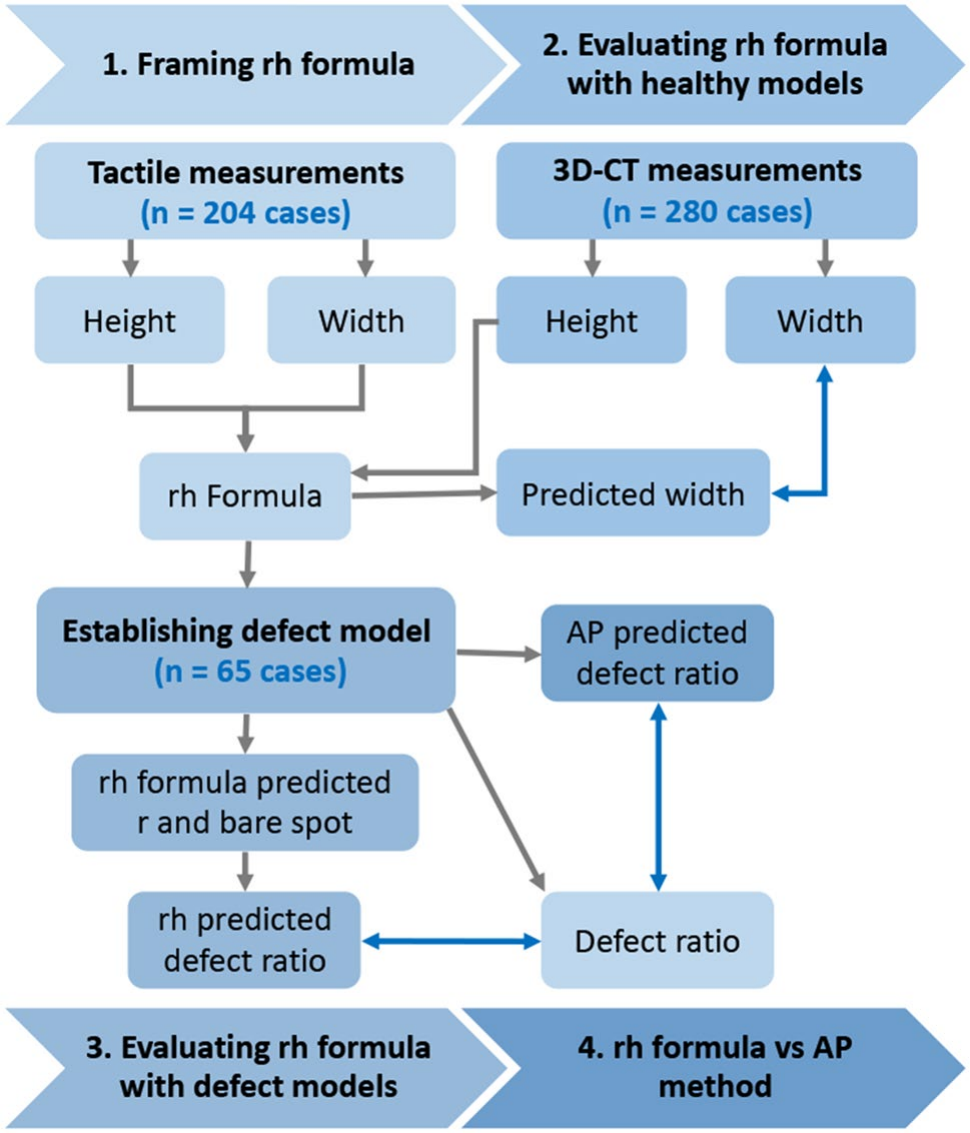
Flow chart of the study design

Three-dimensional (3D) reconstruction computed tomography (CT) helps preoperative treatment decisions in anterior glenohumeral instability [3, 7]. The AP method determines the glenoid defect ratio based on the height and an approximate width perpendicular to the height, and it is now commonly used in clinic [8, 9]. However, recent studies questioned the accuracy of the AP method in quantifying the glenoid bone loss: It overestimates the glenoid bone loss via comparing to the defect ratio determined using the method based on the *en face* area [3, 10, 11]. In this study, a new intuitive method has been framed step by step to quantify glenoid defect via 3D-CT imaging using a self-control design, preoperatively. The aims of this study were to (1) identify a cadaver-based formula to accurately predict the native glenoid width via the glenoid height, i.e. the rh formula, (2) validate the feasibility of the rh formula on CT scans in predicting the native glenoid width, (3) determine the accuracy of rh formula on predicting circle size and quantifying bone loss in the artificially established glenoid defect models and (4) verified its technical superiority via comparing to the AP method (Fig. 1).

## Materials and methods

### Ethics

The donors and their families expressed to willingness for the anatomical research. The inclusion of the glenoid specimens and CT images has been demonstrated in the flow chart (Fig. 1).

### Cadaveric morphometric analysis

**Fig. 2:**
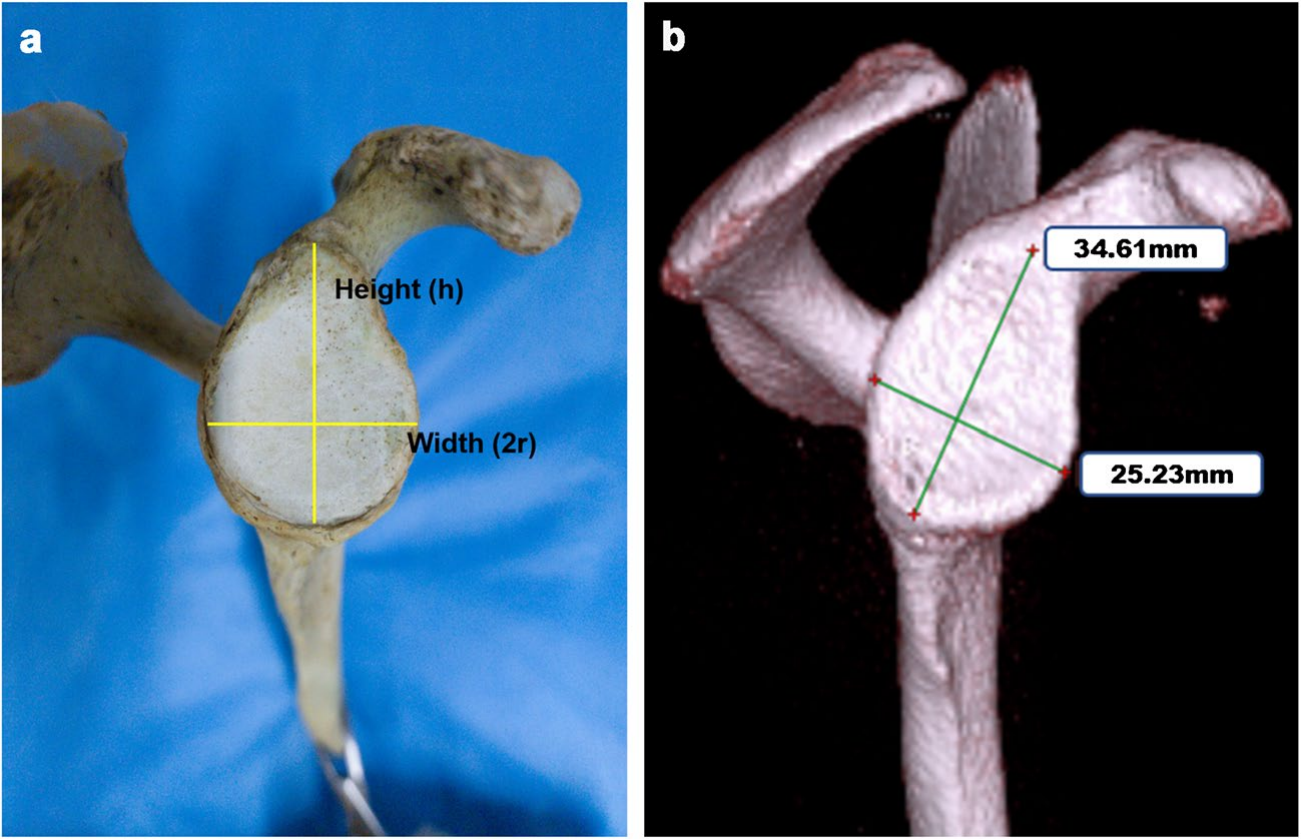
Measurement of the native glenoid height and width. **a** Specimen; **b** 3D-CT image. The glenoid height (*h*) was identified as the longitudinal axis connecting supraglenoid tubercle and the most inferior point. And, the native glenoid width, i.e. the diameter (2*r*) of the best-fit circle, was taken as the distance from the anterior to posterior rims of the glenoid perpendicular to *h*

Tactile measurement (Fig. 2a), including the native glenoid width (2*r*) and height (*h*), was performed on 204 dry cadaveric glenoid with no sign of bone loss, fracture or arthritis using an electronic digital vernier calipre (111–101, Sanliang, Dongguan, China; measurement accuracy ± 0.01 mm). Three orthopaedic surgeons measured the height and width of the intact glenoid. Each parameter was read for three times. The mean of each surgeon was recorded, and the inter-observer reliability was determined. The readings among surgeons were averaged for linear regression.

### Validating the cadaver-based formula on 3D-CT scans

A total of 280 shoulder CT scans with no sign of glenoid defect, multidirectional instability, shoulder fracture or shoulder degenerative diseases were enrolled. The humeral head was carefully eliminated to fully expose the glenoid using RadiAnt DICOM Viewer (version 2022.1., Medixant, Poland). The 3D-CT model was subsequently adjusted to produce an *en face* sagittal view of glenoid (Fig. 2b). One researcher read the height (*h*) and width (2*r*) from the 3D-CT reconstruction images for three times and recorded the means. Another researcher plugged *h* to the *rh* formula to calculate the predicted glenoid width. And, a third surgeon examined the agreement between the calculative glenoid width and the 3D model displayed width.

### Establishing the glenoid defect model

Another 65 3D-CT scans of shoulders were enrolled. The acquired 3D-CT images were used to generate raw DICOM format data. The data were imported into Mimics (version 20.0, Materialise, Belgium) software, and the model was segmented using the threshold segmentation command to extract the complete 3D model of glenoid and export the model in STL format. One researcher measured and recorded the intact height (*h*) and width for further analysis. Subsequently, after importing into Geomagic Wrap (version 2017, Raindrop, USA), the files were saved in STEP format and imported into Solidworks (version 2021, Dassault Systemes, France) for constructing glenoid models.

Attributing to the high incidence in clinic [12, 13], the anterior–inferior glenoid defect models were artificially established for further validation. A randomly generated sequence range 0–0.25 was randomly assigned to the 3D model as the original defect ratio. The ‘split tool’ in the Soildwork software was recruited. The splitting point was pinned based on the original defect ratio and native glenoid width of the model. And, the cutting angle was approximately parallel to the glenoid height and perpendicular to the *en face* of glenoid.

### Evaluating the formula in predicting glenoid defect ratio

**Fig. 3:**
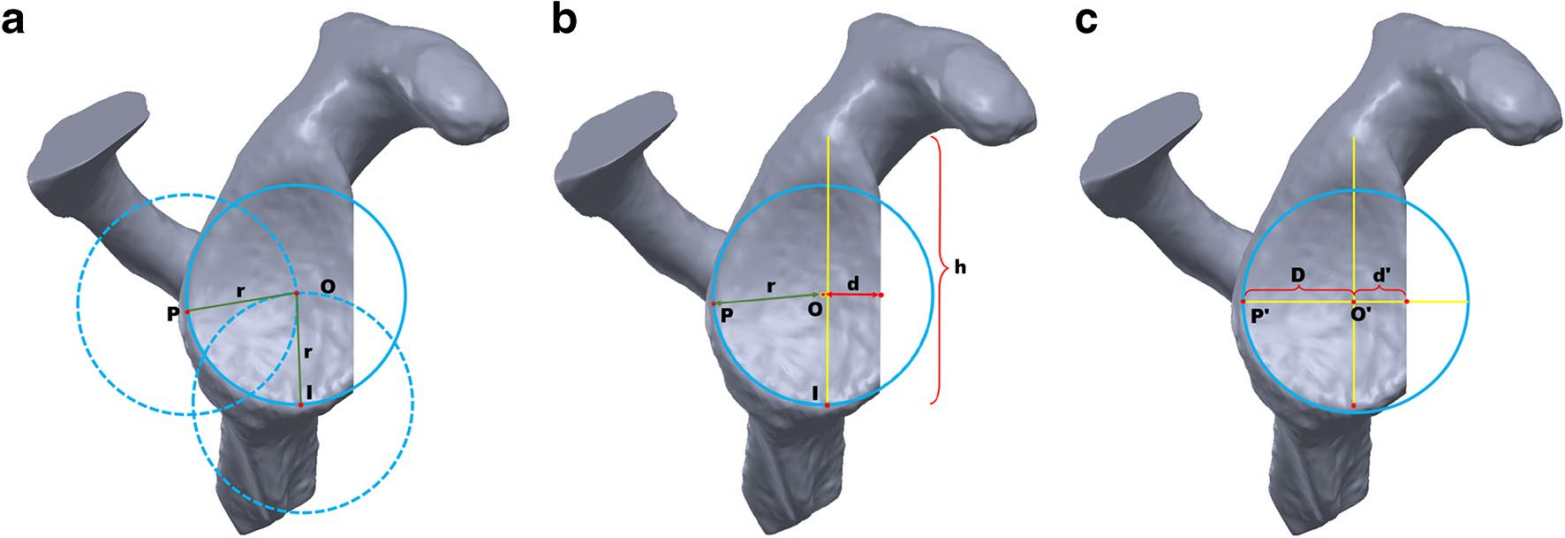
Diagram of rh formula and AP method in calculating defect ratio in artificial established glenoid defect models. The predicted width, *i.e.* the diameter of the approximate best-fit circle (2*r*, **a**, **b**), is obtained by putting the glenoid height (*h*) into the rh formula. **a** The bare spot (O) is located by fitting the circle of known size (full line) tangent with the posterior (P) and inferior (I) rims of the glenoid using Solidworks or redefined as the intersection point of the circles (dashed line) centred at P and I. **b** The defect ratio is calculated as: $\frac{r-d}{2r}$; *d* is the distance from O to the deficient anterior glenoid margin. **c** In AP method, O is redefined as the intersection of *h* and the widest diameter (*D* + *d*) on the *en face* deficient glenoid [9], which are at 90° to each other. *D* is from O to the posterior rim and *d* is from O to the anterior rim. *D* is taken as the radius of the approximate best-fit circle, and the defect ratio is calculated as: $\frac{D-d}{2D}$

A second researcher plugged the *h* value into the rh formula for a predicted glenoid width (2*r*). Thus, the size of the best-fit circle is determined. A circle of fixed size was tangent to posterior (P) and inferior (I) rims of the glenoid using the ‘perimeter circle’ command in Solidworks (Fig. 3a, full line), which located the bare spot in the glenoid fossa. Alternatively, two circles of fixed size were centred at the P and I points, and the intersected point of the circles was considered as the bare spot (Fig. 3a, dotted line).

Then, the distance (*d*) between O and the deficient anterior rim of the glenoid was measured (Fig. 3b). Subsequently, the glenoid defect ratio was calculated using the ratio method as (*r* − *d*)/2*r*. A third researcher examined the agreement between the model parameter and predicted value, including the glenoid width and defect ratio. Moreover, the AP method was employed which took the intersection of the longitudinal axis and widest diameter of the deficient glenoid model as the bare spot (O; Fig. 3c). The second researcher measured the glenoid width (2*D*), calculated the defect ratio ((*D* − *d*)/2*D*) and the third researcher tested the accuracy of AP prediction.

### Statistical analyses

The data were processed using SPSS 25.0 (IBM, Armonk, NY). The outcome parameters were present as mean ± standard division (SD). The intraclass correlation coefficient (*ICC*) was used to detect the inter-observer reliability in tactile measurement and the accuracy of the rh formula and AP method (*ICC* can be interpreted as poor for *ICC* < 0.5, moderate for *ICC* ranged 0.5–0.75, good for *ICC* > 0.75 to 0.9 and excellent for *ICC* > 0.9). A linear regression was calculated to detect the relationship between the intact glenoid height and width, and the determination coefficient (*R*^2^) was used to qualify the linearity and predictability of the glenoid height in the native glenoid width. Moreover, paired *t*-test was employed to identify the difference between the predicted value and model parameter. A *p*-value < 0.05 was deemed significant.

## Results

### A Linear relationship exists between the intact glenoid height and width

**Fig. 4:**
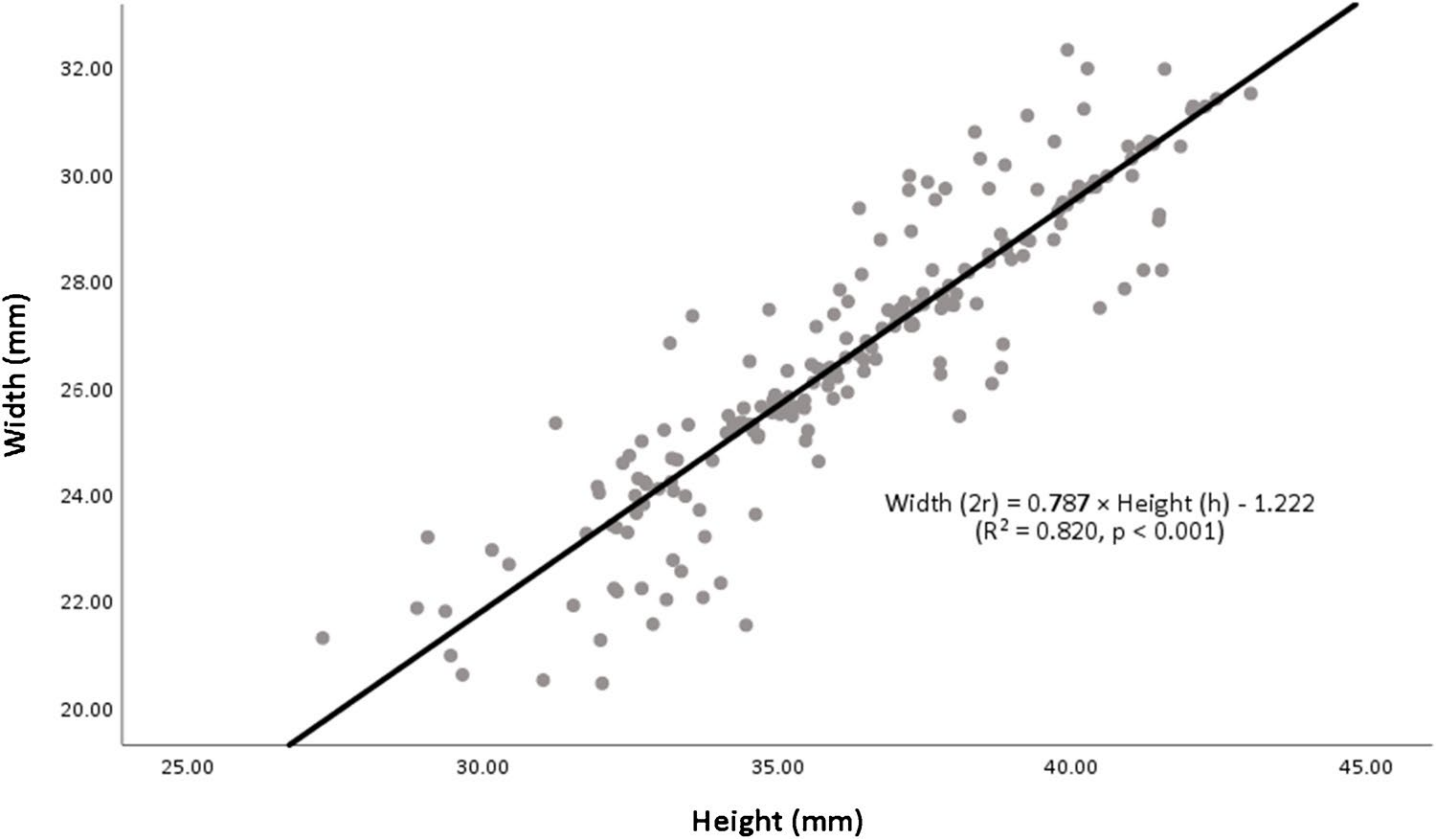
The linear relationship between the native glenoid height and width. The dot represents the glenoid height and width of each specimen. The solid line represents the linear regression based on the cadaver cohort. The rh formula: glenoid width (2*r*) = 0.768 × glenoid height (*h*) − 1.222 mm (*R*.^2^ = 0.820, *p* < 0.001)

The inter-observer reliability was good to excellent (*ICC*_Height_ = 0.935, *ICC*_Width_ = 0.875; Supplemental Table 1). The mean values for the height and width of the glenoid in 204 cases were measured to be 36.31 ± 3.16 mm and 26.66 ± 2.68 mm, respectively (Supplemental Table 1). The linear regression formula (rh formula) was revealed by using the width of the native glenoid as the dependent variable and the height as the independent variable in 204 cases, i.e. the rh formula: width (2*r*) = 0.768 × height (*h*) − 1.222 mm (*R*^2^ = 0.820, *p* < 0.001; Fig. 4).

### The cadaver-based formula validates on 3D-CT images

The glenoid height and width of the 3D-CT image cohort were 36.11 ± 2.31 mm and 26.56 ± 1.96 mm (Supplemental Table 2). Moreover, the calculative glenoid width was 26.51 ± 1.77 mm (Supplemental Table 2). An excellent agreement was found between the model glenoid width and the calculative one (*ICC*_Width_ = 0.911). No statistically significant difference was detected between the model parameter and predicted value (*p* = 0.266). Moreover, in terms of gender and should side, the agreements between model measurement outcome and rh formula–predicted value were good and above, and no difference was found in between (Table 1).

**Table 1:** The accuracy of rh formula in predicting intact glenoid width on 3D-CT images

Subgroup	Category	Model parameter (mm)	Predicted value (mm)	*ICC*	*p*-value
Gender	Male	27.77 ± 1.84	27.78 ± 1.49	0.878	0.896
	Female	25.58 ± 1.44	25.47 ± 1.22	0.842	0.089
Side	Left	26.77 ± 2.06	26.72 ± 1.85	0.927	0.350
	Right	26.32 ± 1.83	26.28 ± 1.65	0.887	0.521

^a^*ICC* intraclass correlation coefficient

### The rh formula qualifies in predicting glenoid bone loss

The glenoid height and width of the intact models were 36.12 ± 3.19 mm and 26.55 ± 2.75 mm, respectively (Supplemental Table 3). Moreover, the rh formula–predicted glenoid width was 26.52 ± 2.45 mm, while the AP method–predicted value was 27.11 ± 2.99 mm (Supplemental Table 3). An excellent agreement was found by comparing the rh formula–predicted width and AP method–predicted width with the original model width (*ICC*_rh_ = 0.967, *ICC*_AP_ = 0.933; Table 2). Statistically significant difference was detected between the AP method–predicted width and model parameter (*p*_AP_ = 0.001) while absent as to rh formula (*p*_rh_ = 0.778; Table 2).

**Table 2:** The accuracy of rh formula and AP method in predicting native glenoid width and defect ratio in artificially established glenoid defect models

	Model parameter (mm)	Method	Predicted value (mm)	*ICC*	*p*-value
Glenoid width (mm)	26.55 ± 2.75	rh formula	26.52 ± 2.45	0.967	0.778
		AP method	27.11 ± 2.99	0.933	0.001
Defect ratio (%)	14.39 ± 5.16	rh formula	14.23 ± 5.24	0.997	0.572
		AP method	15.19 ± 5.36	0.911	0.033

*ICC* intraclass correlation coefficient, *rh* the native glenoid width (2r) and height (h), *AP* anterior–posterior.

The glenoid defect ratio was 14.39 ± 5.16% (Supplemental Table 3). Moreover, the rh formula–predicted defect ratio was 14.23 ± 5.24%, while the AP method–predicted value was 15.19 ± 5.36% (Supplemental Table 3). Excellent agreements were found using the rh formula and AP method (*ICC*_rh_ = 0.997, *ICC*_AP_ = 0.911; Table 2). Statistically significant difference was only detected between the AP method–predicted defect ratio and model parameter (*p*_AP_ = 0.033, *p*_rh_ = 0.572; Table 2).

## Discussion

An international survey at 2016 about surgeon’s preferences in diagnostic work-up towards treating anterior shoulder instability reported no consensus regarding the protocol in assessing glenoid bone loss [6], given the difficulties in determining the native glenoid in the context of glenoid deficiency and redefining the bare spot for the approximate best-fit circle. It has been found in this study that the rh formula was qualified in estimating the native width of glenoid in 3D-CT images of intact glenoid. It predicted the native glenoid width, redefined the bare spot and accurately determined the defect ratio of the artificially established defect model, which is of higher accuracy compared to the clinically common AP method.

Do et al. [14] and Lacheta et al. [5] proposed that best-fit circles based on a deficient glenoid do not always represent the native glenoid and may thus lead to inaccurate circle sizes and defect estimates. Moreover, an inestimable bare spot on *en face* of the deficient glenoid is the limiting factor to the popularization and application of the existed formulas [15–18], though the relationship between glenoid height and width has been explored in studies [19–21]. For example, Giles et al. [21] also found the linear relationship between the glenoid height and width on based on the CT images. However, a cadaver study revealed a suboptimal accuracy of the Giles method with an overestimation of 13.7% [22]. In terms of the Barchilon method [3], identifying the defect ratio based on the *en face* area of the defect glenoid within the best-fit circle, it is not feasible in clinic due to the lack of appropriate area measurement tools in the CT viewers, though an overestimation of less than 2% has been reported in assessing glenoid deficiency [22]. This study proposes a new intuitive way to predict the native glenoid width using the rh formula and redefine the bare spot based on predicted native glenoid width (Fig. 3A). The self-control study confirmed a good accuracy (*p*_rh_ = 0.778) and reproducibility (*ICC*_rh_ > 0.9) of the rh formula in predicting the native width in glenoid deficiency.

In the study, the AP method is inferior to the rh formula in predicting the intact glenoid width and defect ratio by comparing to the model parameters (width, *p*_rh_ = 0.778, *p*_AP_ = 0.001; defect ratio, *p*_rh_ = 0.572, *p*_AP_ = 0.033), respectively. The reasons may be as follows: the intersection of the height and width on the *en face* of glenoid is not equal to the bare spot of the best-fit circle. Paralleled with our findings, previous studies indicate that the various defect ratios are detected at different clock face location using the AP method [7, 23–25]. However, in the rh formula, the diameter of the best-fit circle is solely derived from the glenoid height regardless of the glenoid face location, which overcomes the difficulties.

Limitations exist. The rh formula is developed based on a Chinese cadaver cohort. The population effect and individual difference, e.g. the shape of glenoid, may diminish the predictive effect of rh formula. In this study, the superiority of the rh formula was found by compared with the AP method in determining the diameter of the best-fit circle and defect ratio. To date, the glenoid deficiency evaluating methods stop at verifying the accuracy statistically. However, a cohort study focusing on the postoperative outcome of the bone block procedures with preoperatively or perioperatively estimating the glenoid defect ratio using the rh formula, like Latarjet and Bristow procedures [26, 27], is advocated in the future. Indeed, this intuitive formula takes the glenoid height as the only variable in determining the diameter of the best-fit circle. Further investigation is needed to evaluate if the quick answer from the rh formula is precise enough in predicting glenoid bone loss compared to other formulas bringing into multiple factors, such as the PICO and Shaha methods [28, 29]. The rf formula is designed for unipolar glenoid defects. In terms of the bipolar lesion, such as Hill-Sachs lesions, the rh formula is not qualified and the glenoid track method has been applied in clinic [30, 31].

## Conclusions

The cadaver-based formula, i.e. the rh formula, is valid in 3D-CT images and qualified in predicting glenoid deficiency, which helps preoperative treatment decisions in anterior glenohumeral instability and motivates personalized medicine in orthopaedics. Generally, the flow of this study, i.e. validating the routine obtained from intact specimens in 3D-CT scans to reproduce the native index and predict the severity in deficient state, may motivate the development of new techniques, including but not limited to preoperatively estimating glenoid bone loss.

### Supplementary Information

Below is the link to the electronic supplementary material.

Supplementary file1 (XLSX 26 KB)

Supplementary file2 (XLSX 20 KB)

Supplementary file3 (XLSX 16 KB)

## Data Availability

The authors confirm that the data supporting the findings of this study are available within the supplementary materials.

## References

[1] Willemot LB, Elhassan BT, Verborgt O (2018). Bony reconstruction of the anterior glenoid rim. J Am Acad Orthop Surg.

[2] Klemt C, Toderita D, Nolte D, Di Federico E, Reilly P, Bull AMJ (2019). The critical size of a defect in the glenoid causing anterior instability of the shoulder after a Bankart repair, under physiological joint loading. Bone Joint J.

[3] Barchilon VS, Kotz E, Barchilon Ben-Av M, Glazer E, Nyska M (2008). A simple method for quantitative evaluation of the missing area of the anterior glenoid in anterior instability of the glenohumeral joint. Skeletal Radiol.

[4] Rouleau DM, Garant-Saine L, Canet F, Sandman E, Ménard J, Clément J (2017). Measurement of combined glenoid and Hill-Sachs lesions in anterior shoulder instability. Should Elb.

[5] Lucca L, Elmar H, Andreas V, Sepp B, Pia J, Millett PJ, Andreas I, Frank M (2019). Insufficient consensus regarding circle size and bone loss width using the ratio-”best fit circle”-method even with three-dimensional computed tomography. Knee Surg Sports Traumatol Arthrosc.

[6] Weel H, Tromp W, Krekel PR, Randelli P, van den Bekerom MP, van Deurzen DF (2016). International survey and surgeon’s preferences in diagnostic work-up towards treatment of anterior shoulder instability. Arch Orthop Trauma Surg.

[7] Altan E, Ozbaydar MU, Tonbul M, Yalcin L (2014). Comparison of two different measurement methods to determine glenoid bone defects: area or width?. J Shoulder Elbow Surg.

[8] Kho J, Kholinne E, Lim S, Hong H, Kwak JM, Sun Y, Koh KH, Jeon IH (2019). Arthroscopic bare spot method underestimates true bone defect in bony Bankart lesion. Arch Orthop Trauma Surg.

[9] Provencher MT, Bhatia S, Ghodadra NS, Grumet RC, Bach BR, Dewing CB, LeClere L, Romeo AA (2010). Recurrent shoulder instability: current concepts for evaluation and management of glenoid bone loss. J Bone Joint Surg Am.

[10] Lederman ES, Shah AA (2022). A ratio estimating glenoid bone loss. JSES Int.

[11] Bakshi NK, Cibulas GA, Sekiya JK, Asheesh B (2018). A clinical comparison of linear- and surface area–based methods of measuring glenoid bone loss. Am J Sports Med.

[12] Zacchilli MA, Owens BD (2010). Epidemiology of shoulder dislocations presenting to emergency departments in the United States. J Bone Joint Surg Am.

[13] Hasebroock AW, Joseph B, Lukas F, Bowens JP (2019). Management of primary anterior shoulder dislocations: a narrative review. Sports Med Open.

[14] Sung DW, Hyung KJ, Ryul LJ, Hwan YT, Min CY (2023). Disagreement between the accepted best-fit circle method to calculate bone loss between injured and uninjured shoulders. Am J Sports Med.

[15] Wu YG, Zhang HL, Hao YF, Jiang CY (2019). Reliability of the measurement of glenoid bone defect in anterior shoulder instability. Chin Med J.

[16] Moroder P, Plachel F, Huettner A, Ernstbrunner L, Minkus M, Boehm E, Gerhardt C, Scheibel M (2018). The effect of scapula tilt and best-fit circle placement when measuring glenoid bone loss in shoulder instability patients. Arthroscopy.

[17] Sang-Jin S, Jae JB, Won KY, McGarry MH, Lee TQ (2018). Estimation of anterior glenoid bone loss area using the ratio of bone defect length to the distance from posterior glenoid rim to the centre of the glenoid. Knee Surg Sports Traumatol Arthrosc.

[18] Franz K, Felix A, Stefano L, Michael R, Markus W (2006). Is the bare spot a consistent landmark for shoulder arthroscopy? A study of 20 embalmed glenoids with 3-dimensional computed tomographic reconstruction. Arthroscopy.

[19] Zuo CX, Xi LT, Chen Ying Du, Lei LW, Fang LP (2020). Simple linear calculating method of glenoid bone defects using 3-dimensional computed tomography based on an east Asian population in China. Orthop J Sports Med.

[20] Owens BD, Burns TC, Campbell SE, Svoboda SJ, Cameron KL (2013). Simple method of glenoid bone loss calculation using ipsilateral magnetic resonance imaging. Am J Sports Med.

[21] Giles JW, Owens BD, Athwal GS (2015). Estimating glenoid width for instability-related bone loss: a CT evaluation of an MRI formula. Am J Sports Med.

[22] Arenas-Miquelez A, Dabirrahmani D, Sharma G, Graham PL, Appleyard R, Bokor DJ, Read JW, Piper K, Raniga S (2021). What is the most reliable method of measuring glenoid bone loss in anterior glenohumeral instability? A cadaveric study comparing different measurement techniques for glenoid bone loss. Am J Sports Med.

[23] Provencher MT, Detterline AJ, Ghodadra N, Romeo AA, Bach BR, Cole BJ, Verma N (2008). Measurement of glenoid bone loss: a comparison of measurement error between 45 degrees and 0 degrees bone loss models and with different posterior arthroscopy portal locations. Am J Sports Med.

[24] Lo IK, Parten PM, Burkhart SS (2004). The inverted pear glenoid: an indicator of significant glenoid bone loss. Arthroscopy.

[25] Saito H, Itoi E, Sugaya H, Minagawa H, Yamamoto N, Tuoheti Y (2005). Location of the glenoid defect in shoulders with recurrent anterior dislocation. Am J Sports Med.

[26] Charles B, Christophe T, Michel C, Mehta SS, Pascal B (2014). The open latarjet procedure is more reliable in terms of shoulder stability than arthroscopic bankart repair. Clin Orthop Relat Res.

[27] Helfet AJ (1958). Coracoid transplantation for recurring dislocation of the shoulder. J Bone Joint Surg Br.

[28] Marcello Z, Domenico A, Alberto A, Antonio B, Luca B, Alessandro C, Andrea C, Massimo DF, Francesco DP, Giuseppe DSM, Annibale GE, Salvatore G, Pasquale G, Giovanni M, Carmelo M, Riccardo R, Maria RA, Raffaele R, Michele T, Salvo RP, Maria SL, Vito C (2023). Glenoid bone loss in anterior shoulder dislocation: a multicentric study to assess the most reliable imaging method. Radiol Med.

[29] Shaha JS, Cook JB, Song DJ, Rowles DJ, Bottoni CR, Shaha SH, Tokish JM (2015). Redefining “critical” bone loss in shoulder instability: functional outcomes worsen with “subcritical” bone loss. Am J Sports Med.

[30] Yamamoto N, Itoi E, Abe H, Minagawa H, Seki N, Shimada Y, Okada K (2007). Contact between the glenoid and the humeral head in abduction, external rotation, and horizontal extension: a new concept of glenoid track. J Shoulder Elbow Surg.

[31] Kawakami J, Yamamoto N, Etoh T, Hatta T, Mineta M, Itoi E, Isawa R (2019). In vivo glenoid track width can be better predicted with the use of shoulder horizontal extension angle. Am J Sports Med.

